# Identifying risk factors for *Plasmodium* infection and anaemia in Kinshasa, Democratic Republic of Congo

**DOI:** 10.1186/s12936-016-1412-5

**Published:** 2016-07-15

**Authors:** Giovanfrancesco Ferrari, Henry M. T. Ntuku, Amanda Ross, Sandro Schmidlin, Didier M. Kalemwa, Antoinette K. Tshefu, Christian Lengeler

**Affiliations:** Swiss Tropical and Public Health Institute, P.O. Box, 4002, Basel, Switzerland; University of Basel, Basel, Switzerland; Kinshasa School of Public Health, Kinshasa, Democratic Republic of the Congo

**Keywords:** Malaria, Malaria risk, Anaemia, Fever, ITN use, ITN ownership, Democratic Republic of Congo, Kinshasa

## Abstract

**Background:**

There is little data on the risk factors for malaria infection in large cities in central Africa and in all age groups. There may be different associations with the risk factors for areas with different malaria transmission intensities such as the effect of fever or age. This study aimed at identifying risk factors associated with *Plasmodium* infection and anaemia among children 6–59 months and individuals aged older than 5 years in Kinshasa, a large city with heterogeneity in malaria prevalence.

**Methods:**

This study analysed data from 3342 children aged 6–59 months from 25 non-rural health zones (HZs) and for 816 individuals aged older than 5 years from two HZs in Kinshasa (non-rural), collected during a cross sectional malaria survey in 2011. Logistic regression with random effects was used to investigate predictors for malaria and anaemia. Differences in risk factors in areas with a prevalence of less than 10 and 10 % or greater were investigated.

**Results:**

There was evidence of a different age-pattern in the two transmission settings. For children under 5 years, the highest prevalence of malaria was observed in the 48–59 months group in both transmission settings, but it increased more gently for the lower transmission HZs (p = 0.009). In a separate analysis in children over 5 years in two selected HZs, the peak prevalence was in 5–9 years old in the higher transmission setting and in 15–19 years old in the lower transmission setting. Reported fever was associated with malaria in both transmission strata, with no evidence of a difference in these associations (p = 0.71); however in children older than 5 years there was a significant interaction with a stronger association in the low transmission HZ. Insecticide-treated net (ITN) use was associated with a lower risk of malaria infection in children 6–59 months in the high transmission HZs. Similar estimates were found in children over 5 years and the lower transmission HZ but the associations there were not significant. There was no evidence of a difference in these associations by strata. The risk of anaemia decreased with increasing age in all strata, whereas it increased with malaria infection and reported fever. ITN use did not show evidence of protection against anaemia. Low socio-economic status was associated with malaria in high transmission setting in children 6–59 months and anaemia in low transmission setting.

**Conclusions:**

This study shows that in areas of low transmission in Kinshasa, the peak prevalence occurs in older age groups however ITN use was highest in children under 5 years. Targeted distribution of ITN to all age groups should be continued. For most risk factors, there was no evidence of an interaction with transmission intensity however the associations with age and with fever in the last 2 weeks did vary significantly.

## Background

Urbanization, widespread use of malaria control measures and effective treatment in recent years have had a significant impact in reducing the prevalence of malaria in many African cities, as well as contributing to the more heterogeneous risk in malaria observed in many urban areas [1–5]. In Kinshasa, the capital of the DRC malaria has considerably decreased during the past 30 years. A survey conducted in 2011, showed a prevalence of 17 % among children 6–59 months of age, and the existence of a gradient of prevalence from the centre (lower risk) to the periphery (higher risk) [6–9]. Moreover, traditionally attention has focused on high risk groups, and little attention has been put on older children and adults. Understanding the complex heterogeneity of risk factors that can contribute to increased risk of malaria in urban settings across different age groups will enable a more effective use of control measures.

In addition, many studies have shown that control of malaria can reduce the prevalence of childhood anaemia [10–14]. Anaemia, in particular due to iron deficiency, is a major public health challenge in paediatric populations in sub-Saharan Africa, and in DRC it is among the top five leading causes of years lived with disability (YLDs) (GBD 2010). In Kinshasa the current rate of anaemia (Hb < 11 g/dl) among pre-school aged children was 65 % in 2011 [6].

The present study investigated individual and household risk factors for *Plasmodium falciparum* infection and anaemia in Kinshasa in children aged 6–59 months in 25 non-rural HZs, as well as in individuals aged 5 years and older in a separate analysis in two HZs.

## Methods

### Study area and recruitment of study participants

This study used cross-sectional data from a survey conducted in 2011 in Kinshasa, the capital city of the DRC, which is described in detail by Ferrari et al. [6]. In summary, data collection took place from April to June 2011, before the end of the rainy season and included 2512 households selected through a multi stage sampling procedure to obtain a sample of 3342 children aged 6–59 months from 25 health zones (HZs) and 816 individuals aged 5 years or older from two HZs selected out of the 25 (Ngiri Ngiri and Selembao). The HZ represents the primary operational unit of the health system in DRC, and covers about 150,000 inhabitants. It includes a general referral hospital, health centres and lower-level health facilities. Each HZ is further divided in health areas. In Kinshasa malaria transmission is ensured by *Anopheles gambiae s.l.*, and usually peaks during the long rainy season from September to May [15]. From each participant a finger-prick blood sample was collected to test for malaria by rapid diagnostic test (RDT) (SD Bioline Malaria Antigen P.f/Pan), providing an immediate on-site diagnosis. The level of haemoglobin (Hb) was measured with a HemoCue 201 plus + photometer (Ångelholm, Sweden). Axillary temperature was measured using a digital thermometer and the individual’s history of fever in the preceding 2 weeks was also recorded. A standardized electronic survey questionnaire was administered to all heads of eligible household using an HTC smartphone running with Android OS. The survey questionnaire was an adaptation of the standard malaria indicator survey questionnaire from the Roll Back Malaria Partnership (http://www.RBM.org) created with the Build component of the open data kit (ODK) software (University of Washington & Google Foundation). Respondents were asked about demographic information of the residents, educational level, assets owned (such as television and bicycle), presence of insecticide-treated bed net (ITN) and use of ITN the night prior to the survey.

### Assessing risk factors of *Plasmodium* infection and anaemia

The analysis was stratified according to malaria transmission intensity, based on the prevalence of malaria infection measured in 2011 among children 6–59 months [6]. The prevalence ranged from 0.7 to 46 % in children aged 6–59 months. Two strata were defined at the HZ level: a prevalence of infection below 10 % or a prevalence above 10 %. The 10 % prevalence cut-off was an arbitrary selection to allow enough observations in each strata. *Plasmodium* infection and anaemia were assessed for their association with a number of variables. For individuals aged older than 5 years, data collection took place in only two HZs with different transmission intensities (Ngiri Ngiri, 0.8 % and Selembao, 26.8 % in children younger than 5 years); these data were analysed separately.

The primary outcomes of the study were the presence or absence of *Plasmodium* malaria as measured by rapid diagnostic test (RDT) and the anaemia test results. A child aged between 6 and 59 months was defined as anaemic if his/her Hb was below 11.0 g/dl. Therefore, the outcomes variables were dichotomous. Recorded explanatory variables were: age, gender, educational level of the respondent, occupation of the respondent, insecticide mosquito-net use and reported fever during the last 2 weeks and wealth index. A wealth index, calculated according to the method of Filmer et al., was constructed for each household based on ownership of household assets (having a television, a radio, etc.) and house characteristics (having electricity, drinking water, toilet type, roof and ground material) [16]. Three categories were generated to classify households ranging from the poorest to the least poor in the community.

### Statistical methods

The proportions with malaria infection and with anaemia were analysed using a logistic regression model with random effects to take account clustering by health zone and health area. All analysis were performed separately for children (6–59 months) and individuals older than 5 years since they were sampled from different HZs. The analysis was carried out using STATA version 13 (Stata Corporation College Station, TX, USA).

## Results

Data collection took place in 2512 households, in the 25 HZs that were visited. A total of 3342 children aged 6–59 months were included in the analysis, 1118 and 2224 in the low and high transmission settings, respectively. A similar number of males (50 %) and females were included; the median age was 30 months (90 % central range 9–55). Table 1 shows the number of children examined, by HZ and by transmission strata. For individuals above 5 years of age, data collection took place in two HZs only and included 816 individuals, of which 34 % were males and the median age was 22 years (90 % central range 6–62).

**Table 1:** Number of children 6–59 months examined and the prevalence of *Plasmodium* spp. in Kinshasa, by health zone and strata, 2011

Health zone	Malaria prevalence in children aged 6–59 months [95 % CI]					
	<10 %			>10 %		
	%		N	%		N
Bandalungwa	1.5	[0.2–5.3]	134			
Barumbu	2.4	[0.5–6.9]	125			
Binza Météo				24.8	[17.0–34.0]	109
Binza Ozone				19.1	[12.9–26.7]	136
Biyela				46.0	[37.1–55.1]	126
Gombe				11.5	[6.7–18.0]	139
Kalamu I				16.2	[8.4–27.1]	68
Kalamu II	2.5	[0.8–5.7]	200			
Kikimi				32.8	[24.9–41.6]	131
Kimbanseke				36.1	[27.9–44.9]	133
Kingasani				25.0	[18.3–32.7]	152
Kinshasa	0.7	[0.0–4.0]	136			
Kintambo				11.7	[7.0–18.1]	145
Lemba	7.7	[3.8–13.7]	130			
Limete				17.3	[11.3–24.8]	133
Lingwala	0.7	[0.0–4.1]	135			
Makala				17.9	[11.8–25.5]	134
Masina I				12.3	[7.3–19.0]	138
Masina II				24.8	[17.7–33.0]	133
Mont Ngafula I				33.6	[25.7–42.2]	134
Mont Ngafula II				35.3	[27.3–44.1]	133
Ngaba	7.5	[3.6–13.3]	134			
Ngiri Ngiri	0.8	[0.0–4.2]	124			
Police				17.0	[11.1–24.5]	135
Selembao				26.8	[19.9–34.7]	145
Total N			1118			2224

### Risk factors for *Plasmodium* infection in children aged 6–59 months (25 HZs)

The risk factors for *Plasmodium* infections in children 6–59 months are shown in Table 2. There was an increase in the proportion with malaria infection with age in both transmission strata. The greatest risk was in children 48–59 months: an odds ratio (OR) of 5.86 [95 % confidence interval (CI) 1.62–21.17] for the 36–47 months group and an OR of 15.53 (95 % CI 4.26–56.64) for the 48–59 months group, compared to the youngest age group. The effect was also seen in higher transmission strata, although the OR was lower: an OR of 1.73 (95 % CI 1.36–2.20) for the 36–47 months group and an OR of 2.54 (95 % CI 1.93–3.35) for the 48–59 months group compared to the youngest age group. The interaction between age and transmission intensity was significant (p = 0.009).

**Table 2:** Univariate and multivariable analysis of risk factors associated with malaria in children between 6 and 59 months of age in Kinshasa, stratified by malaria transmission zone, 2011

			<10 % prevalence								>10 % prevalence						Interaction by transmission zone
			Univariate analysis			Multivariate analysis					Univariate analysis			Multivariate analysis			
Variable	*n*	(%)	OR	95 % CI	p value	OR	95 % CI	p value	*n*	(%)	OR	95 % CI	p value	OR	95 % CI	p value	p value
*Sex*																	
Male	521	3.1	1			1			1141	23.6	1			1			
Female	527	2.7	0.86	0.42–1.8	0.687	0.87	0.41–1.88	0.731	1162	23.3	1.01	0.84–1.23	0.886	0.98	0.80–1.21	0.857	0.670
*Age (months)*																	
6–35	446	0.7	1			1			958	17.8	1			1			
36–47	416	3.1	4.76	1.35–16–84		5.86	1.62–21.17		872	25.5	1.57	1.26–1.97		1.73	1.36–2.20		
48–59	186	7.5	12.02	3.41–42.34	<0.001	15.53	4.26–56.64	<0.001	473	31.1	2.08	1.61–2.68	<0.001	2.54	1.93–3.35	<0.001	0.009
*Reported treated bed net use*																	
No	446	3.6	1			1			1342	27.6	1			1			
Yes	596	2.3	0.65	0.31–1.34	0.240	0.82	0.38–1.76	0.606	961	17.6	0.56	0.46–0.69	<0.001	0.62	0.50–0.77	<0.001	0.705
*Fever in the last 2* *weeks*																	
No	798	1.6	1			1			1744	18.3	1			1			
Yes	245	6.9	4.50	2.15–9.41	<0.001	5.53	2.52–12.11	<0.001	559	39.4	2.89	2.34–3.56	<0.001	2.94	2.36–3.68	<0.001	0.254
*Education of the respondent*																	
No education	24	8.3	1			1			220	32.3	1			1			
Primary	390	3.6	0.41	0.09–1.92		0.35	0.07–1.82		1135	26.5	0.76	0.55–1.03		0.90	0.65–1.26		
Secondary	471	2.8	0.31	0.07–1.47		0.28	0.05–1.50		740	19.7	0.52	0.37–0.72		0.78	0.54–1.14		
Superior and above	163	0.6	0.07	0.01–0.78	0.080	0.05	0.00–0.68	0.084	208	10.6	0.25	0.15–0.42	< 0.001	0.47	0.26–0.86	0.056	0.754
*Occupation of the respondent*																	
Without occupation	720	2.8	1			1			1523	23.2	1			1			
Manual labour	86	2.3	0.83	0.19–3.63		0.98	0.21–4.47		212	27.8	1.27	0.92–1.76		1.29	0.91–1.84		
Self employed	104	3.8	1.40	0.47–4.18		1.58	0.49–5.11		275	24.7	1.08	0.80–1.46		1.01	0.74–1.38	0.236	
Employed	138	2.9	1.04	0.35–3.11	0.931	1.79	0.51–6.31	0.742	293	20.1	0.83	0.61–1.13	0.229	1.35	0.95–1.94		0.860
*Wealth tertile*																	
Poorest	196	4.1	1			1			1175	31.6	1						
Middle	298	3.0	0.73	0.28–1.93		0.72	0.26–2.04		575	19.5	0.52	0.41–0.67		0.54	0.42–0.70		
Wealthiest	546	2.4	0.57	0.23–1.40	0.488	0.82	0.31–2.13	0.828	540	9.44	0.23	0.17–0.31	<0.001	0.27	0.20–0.38	<0.001	0.142

Treated net use was found to significantly lower malaria infection risk in the higher transmission strata with 38 % protection (OR = 0.62, 95 % CI 0.50–0.77), however the effect was not significant in the lower transmission strata. Children who reported fever in the last 2 weeks had a significantly elevated risk of malaria infection in both strata.

Higher education levels showed a trend towards being protective in both transmission settings (Table 2). However there was no evidence of an association with the occupation of the respondent. Finally, children living in the wealthiest tertile were significantly less likely to have a malaria infection compared to the children from the poorest tertile in strata of high transmission (OR = 0.27, 95 % CI 0.20–0.38, p < 0.001). No evidence was found in the HZs with less than 10 % prevalence (OR = 0.82, 95 % CI 0.31–2.13, p = 0.83), however the interaction between socioeconomic status and transmission was not significant (p = 0.14).

### Risk factors for *Plasmodium* infection in individuals older than 5 years (2 HZs)

The risk factors for *Plasmodium* infection in individuals aged older than 5 years are shown in Table 3. The association between age and malaria infection was strong. The highest prevalence was observed in the 15–19 years age group in the low transmission HZ of Ngiri Ngiri with an OR of 7.11 (95 % CI 1.17–43.05) compared to the 5–9 years-old. In the higher transmission HZ of Selembao however, ORs were lower and more homogeneously distributed across all age groups, compared to the 5–9 years-old group which showed the highest prevalence. The interaction between age and transmission intensity however was not significant (p = 0.11).

**Table 3:** Univariate and multivariable analysis of risk factors associated with malaria in individuals aged >5 years in Kinshasa, stratified by malaria transmission zone, 2011

			Ngiri Ngiri: 0.8 % prevalence								Selembao: 26.8 % prevalence						Interaction by transmission zone
			Univariate analysis			Multivariate analysis					Univariate analysis			Multivariate analysis			
Variable	*n*	(%)	OR	95 % CI	p value	OR	95 % CI	p value	*n*	(%)	OR	95 % CI	p value	OR	95 % CI	p value	p value
*Sex*																	
Male	142	5.6	1.0			1.0			143	28.7	1.0			1.0			
Female	257	4.3	0.75	0.29–1.91	0.548	0.74	0.23–2.37	0.616	274	20.1	0.62	0.39–1.00	0.050	0.66	0.40–1.08	0.102	0.733
*Age*																	
5–9 years	62	4.8	1.0			1.0			76	34.2	1.0			1.0			
10–14 years	68	1.5	0.29	0.03–2.90		0.22	0.01–3.39		68	25.0	0.64	0.31–1.32		0.79	0.37–1.72		
15–19 years	48	14.6	3.36	0.82–13.75		7.11	1.17–43.05		46	28.3	0.76	0.34–1.68		0.85	0.37–1.96		
>20	221	3.6	0.74	0.19–2.87	0.022	1.09	0.21–5.72	0.009	227	17.6	0.41	0.23–0.74	0.021	0.45	0.24–0.83	0.042	0.105
*Reported treated bed net use*																	
No	244	6.1	1.0			1.0			315	25.7	1.0			1.0			
Yes	155	2.6	0.40	0.13–1.24	0.089	0.33	0.09–1.21	0.075	102	14.7	0.50	0.27–0.91	0.017	0.57	0.30–1.09	0.078	0.746
*Fever in the last two weeks*																	
No	366	2.2	1.0			1.0			361	21.6	1.0			1.0			
Yes	33	33.3	22.38	8.17–61.27	<0.001	38.71	11.08–135.23	< 0.001	54	33.3	1.81	0.98–3.37	0.066	2.05	1.07–3.95	0.036	<0.001
*Education of the respondent*																	
No education	8	12.5	1.0			1.0			35	34.3	1.0			1.0			
Primary	120	5.0	0.37	0.04–3.50		0.18	0.01–2.49		186	26.9	0.70	0.33–1.52		0.82	0.36–1.87		
Secondary	189	4.8	0.35	0.04–3.16		0.27	0.02–3.48		145	18.6	0.44	0.19–0.99		0.49	0.20–1.19		
Superior and above	82	3.7	0.27	0.02–2.91	0.802	0.17	0.01–2.87	0.647	51	13.7	0.30	0.11–0.88	0.041	0.32	0.09–1.13	0.115	0.865
*Occupation of the respondent*																	
Without occupation	244	5.7	1.0			1.0			200	26.0	1.0			1.0			
Manual labourer	37	2.7	0.46	0.06–3.58		0.82	0.09–7.91		64	23.4	0.87	0.45–1.68		1.17	0.58–2.37		
Self employed	27	3.7	0.63	0.08–5.00		1.33	0.13–13.64		56	16.1	0.55	0.25–1.19	0.398	0.56	0.24–1.28	0.425	
Employed	91	3.3	0.56	0.16–2.00	0.696	0.59	0.11–3.20	0.913	97	20.6	0.74	0.41–1.33		1.12	0.56–2.23		0.911
*Wealth tertile*																	
Poorest and middle^a^	162	4.9	1.0			1.0			201	25.4	1.0			1.0			
Wealthiest	237	4.6	0.94	0.37–2.38	0.891	1.31	0.18–9.64	0.618	99	19.2	0.74	0.42–1.30	0.293	0.91	0.48–1.73	0.779	0.676

^a^Combined due to low number of observations

ITN use was not found to significantly lower the prevalence of malaria infection, although the estimates were in the direction of being protective. Individuals aged 5 years and older who reported fever in the last 2 weeks had an elevated risk of having malaria infection in both sites, and the association was stronger for the lower transmission: OR = 38.71 (95 % CI 11.08–135.23), and OR = 2.05 (95 % CI 1.07–3.95) in Selembao, with a highly significant interaction term (p < 0.0001). There was no evidence of an effect of higher education levels, occupation of the respondent or socio-economic status.

### Risk factors for anaemia in children aged 6–59 months (25 HZs)

The risk of having anaemia was found to decline progressively with increasing age (Table 4) in both low and high transmission strata (p < 0.001). Although there was no evidence that malaria infection increased the risk of having anaemia in the low transmission strata (OR = 2.01, 95 % CI 0.89–4.51), this effect was significant in the higher transmission strata (OR = 3.40, 95 % CI  2.60–4.44). There was no evidence that reported ITN use was protective for the anaemia status in either strata. There was also no evidence of an association with fever, nor with education or occupation. Belonging to the wealthiest tertile was borderline significantly associated with the risk of having anaemia in both low transmission (OR 0.68, 95 % CI 0.47–0.99) and high transmission strata.

**Table 4:** Univariate and multivariable analysis of risk factors associated with anaemia in children between 6 and 59 months of age Kinshasa, stratified by malaria transmission zone, 2011

Variable	<10 %								>10 %								Interaction by transmission zone
	*n*	(%)	Univariate analysis			Multivariate analysis			*n*	(%)	Univariate analysis			Multivariate analysis			
			OR	95 % CI	p value	OR	95 % CI	p value			OR	95 % CI	p value	OR	95 % CI	p value	p value
*Sex*																	
Male	521	55.5	1.0			1.0			1161	69.0	1.0			1.0			
Female	526	59.7	1.19	0.93–1.52	0.167	1.21	0.93–1.58	0.160	1142	66.5	0.89	0.75–1.07	0.210	0.93	0.77–1.12	0.355	0.064
*Age (months)*																	
6–35	445	73.0	1.0			1.0			957	80.6	1.0			1.0			
36–47	416	50.5	0.38	0.28–0.50		0.38	0.28–0.51		873	61.6	0.39	0.31–0.48		0.35	0.28–0.43		
48–59	186	36.6	0.21	0.15–0.31	<0.001	0.19	0.13–0.28	<0.001	473	53.3	0.28	0.22–0.35	< 0.001	0.23	0.18–0.29	<0.001	0.473
*Education of the respondent*																	
No education	24	70.8	1.0			1.0			220	75.0	1.0			1.0			
Primary	390	62.1	0.67	0.27–1.66		0.64	0.24–1.70		1135	69.4	0.76	0.54–1.05		0.83	0.58–1.20		
Secondary	471	57.7	0.56	0.23–1.38		0.57	0.21–1.50		740	64.9	0.62	0.44–0.86		0.78	0.53–1.15		
Superior and above	162	44.4	0.33	0.13–0.84	<0.001	0.35	0.13–0.99	0.037	208	61.5	0.53	0.35–0.81	0.004	0.80	0.49–1.31	0.629	0.412
*Occupation of the respondent*																	
Without occupation	720	60.7	1.0			1.0			1523	68.8	1.0			1.0			
Manual labourer	86	39.5	0.42	0.27–0.67		0.41	0.25–0.68		212	69.3	1.03	0.75–1.40		0.97	0.69–1.36		
Self–employed	104	60.6	1.00	0.65–1.52		1.03	0.65–1.63		275	65.1	0.85	0.64–1.11		0.77	0.58–1.03		0.010
Employed	137	50.4	0.66	0.46–0.95	<0.001	0.94	0.61–1.44	0.005	293	63.8	0.80	0.62–1.04	0.268	0.92	0.68–1.24	0.313	
*Net use*																	
No	446	57.2	1.0			1.0			1341	68.3	1.0			1.0			
Yes	595	57.5	1.01	0.79–1.30	0.922	0.91	0.70–1.20	0.515	962	67.0	0.94	0.79–1.13	0.524	1.09	0.90–1.32	0.512	0.653
*Malaria infection*																	
No	1017	57.3	1.0			1.0			1762	63.1	1.0			1.0			
Yes	30	66.7	1.49	0.69–3.21	0.302	2.01	0.89–4.51	0.078	540	83.1	2.89	2.26–3.69	<0.001	3.40	2.60–4.44	<0.001	0.119
*Fever in the last 2* *weeks*																	
No	798	54.6	1.0			1.0			1744	65.0	1.0			1.0			
Yes	244	66.4	1.64	1.22–2.21	<0.001	1.30	0.93–1.80	0.197	559	76.4	1.74	1.40–2.17	< 0.001	1.32	1.04–1.67	0.039	0.755
*Wealth tertile*																	
Poorest	196	62.2	1.0			1.0			1176	72.9	1.0			1.0			
Middle	298	65.8	1.17	0.80–1.70	0.423	1.14	0.76–1.71		574	64.1	0.66	0.54–0.82	0.000	0.78	0.62–0.99		
Wealthiest	545	51.9	0.66	0.47–0.92	0.013	0.68	0.47–0.99	0.003	540	60.7	0.58	0.46–0.71	0.000	0.77	0.60–0.99	0.073	0.022

### ITN use

**Fig. 1:**
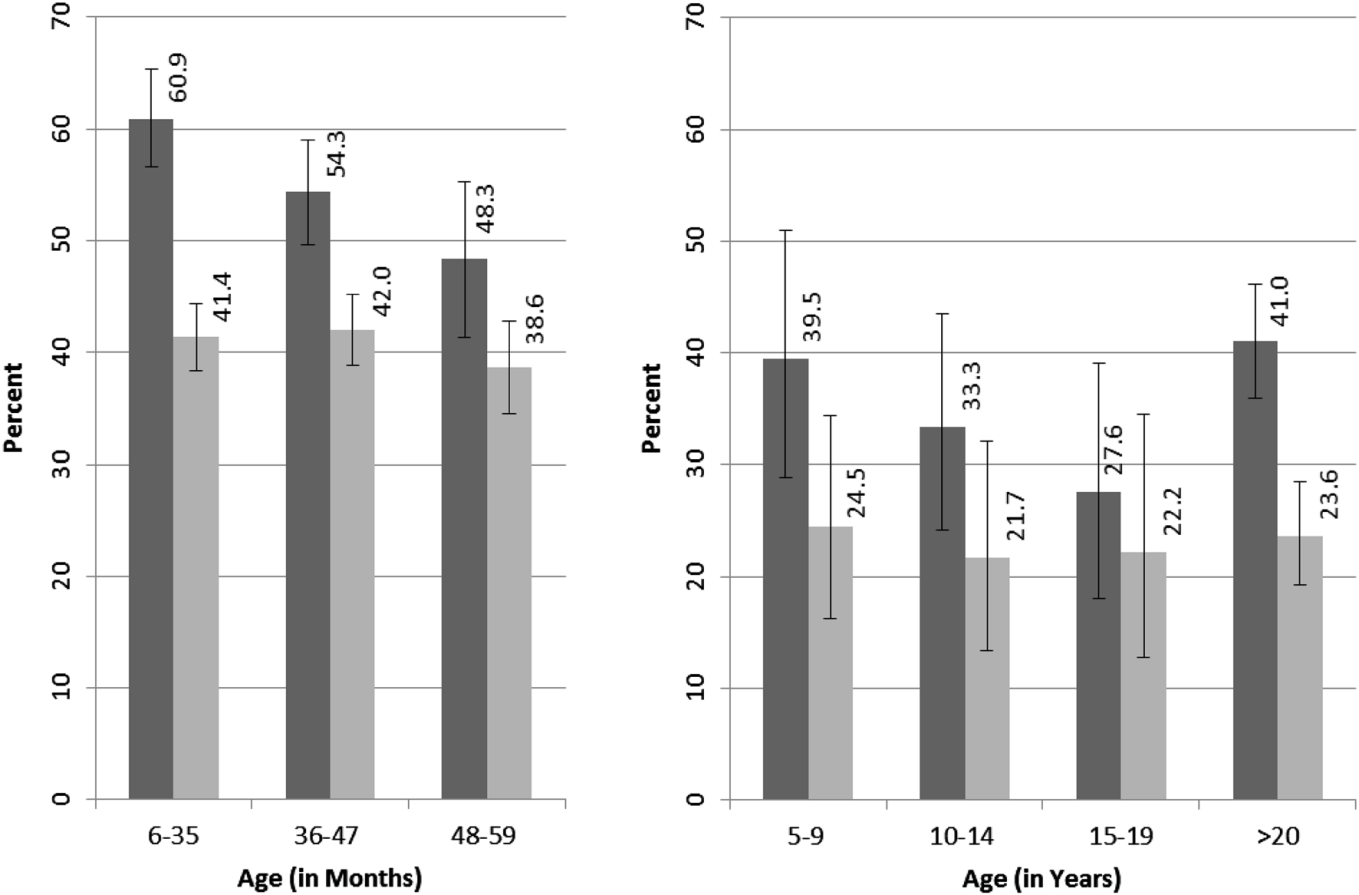
*Left panel* ITN usage among children 6–59 months by transmission intensity. *Right panel* ITN usage among individuals older than 5 years in Ngiri Ngiri (0.8 %) and Selembao (26.8 %) in Kinshasa, 2011. *Light grey bars* represent proportions of reported ITN usage in high transmission strata, and *dark grey bars* in low transmission strata. *Error bars* indicate 95 % CI

There were some age-specific differences in ITN usage (Fig. 1), with highest use in younger children (p = 0.006) in the low transmission strata. In areas of high transmission, ITN usage although lower appeared more homogeneously distributed across age groups (Fig. 1). No significant differences in the utilization were found among individuals age more than 5 years, in both low and high transmission strata (Fig. 1).

## Discussion

The identification of risk factors for malaria infection and anaemia, provides information on the local malaria epidemiology and has the potential to lead to a more effective and targeted use of malaria control measures. This study presents the results of an analysis of the association of a number of variables that alone or in combination could affect the risk of acquiring *Plasmodium* infection and anaemia, in a city with diverse malaria transmission patterns. The key results from this analysis are the association between malaria infection and age, with older age groups being exposed to higher risk of malaria in low transmission settings and a lower and more homogeneous risk across all age groups in high transmission settings. Shift in the age of peak prevalence towards the older groups has been described for malaria and other infectious diseases and is consistent with exposure-related acquired immunity [17, 18]. In zones of low transmission, children are less exposed to infective malaria, hence delaying the age of the first infection and the acquisition of immunity [19]. Clearly, in Kinshasa the risk of *Plasmodium* infection seems to occur later in childhood, which is consistent with areas of rather low levels of transmission. The prevalence rates by HZ shown in Table 1 (range: 0.7–46.0), with most HZs below 30 % confirm that Kinshasa overall has a moderate endemicity level. Recent school surveys done in Kimbanseke, a relatively high prevalence HZ southeast of Kinshasa, found similar results, showing children aged 10–13 at higher risk of malaria and a high prevalence of asymptomatic infections [20].

The relationship of ITN use by the different age groups could also influence the age pattern of risk that was observed: children in the youngest age group, 6–35 months, were significantly more likely to sleep under an ITN in the low transmission setting, whereas utilization was similar among age groups in high transmission setting. A similar shift in age of peak of prevalence towards the older children has been observed elsewhere with an increase in net coverage [21]. Only 44 % of children 6–59 months used an ITN the night preceding the survey, which is still far from universal coverage. In individuals >5 years, overall ITN use was even lower, with only 38 and 23.3 % using an ITN in Ngiri Ngiri and Selembao, respectively. In addition, less than 58 % of HHs owned enough ITNs to cover all household members in 2011. These low values are of concern.

Higher malaria prevalence in older children has also been attributed to increased use of anti-malarials in early childhood [22]. In case of fever in Kinshasa, it is common practice by the caregivers to initially treat their child at home (54 % of the cases) although only 4.3 % of the children treated for fever receive a recommended combination therapy containing artemisinin (unpublished data).

Results indicated that sleeping under an ITN the previous night reduced the risk of *Plasmodium* infection by 38 % (OR 0.62, 95 % CI 0.50–0.77) among children 6-59 months of age in areas of high transmission, consistent with the vast body of evidence supporting the efficacy and effectiveness of ITN in protecting against malaria [11]. In low transmission areas, however, there was no evidence of such an association, presumably because the overall risk of infection was lower.

Reported history of fever was associated with malaria infection overall. There was evidence of a difference in this association with transmission level among individuals aged older than 5 years (p < 0.001). The weaker association of reported fever with malaria in areas of high transmission could be explained by differences in the levels of acquired immunity.

The data confirm that anaemia is frequent in urban Kinshasa, with 65 % prevalence among children 6–59 months, 30 % moderate (7.0–9.9 g/dl) and 1.9 % severe (<7.0 g/dl). ITN use in Kinshasa did not appear to be associated with benefits in lower anaemia risk, contrary to what has been documented in other settings [10–14]. These findings are consistent with a Kenyan study that found only a small difference in prevalence of anaemia between villages with and without ITNs [23]. Anaemia has many causes in addition to malaria [nutrition [24], soil transmitted infections (STH) and schistosomes], and in Kinshasa these are likely to also be contributors to this morbidity. A recent study revealed a high prevalence of STH infections among primary school children in Kinshasa (32.8 %) [20]. Nevertheless, the estimated odds of anaemia in zones of high transmission were 3.5 times (95 % CI 2.70–4.62) higher in malaria infected children.

In this study, the risk of anaemia was shown to decrease with increasing age in both low and high transmission strata. These results are consistent with studies conducted in West Africa, showing a significant reduction in the mean haemoglobin level in children aged 2–5 years compared to children aged 1–2 [25].

This study also showed differences in the effect in malaria risk or anaemia risk by socioeconomic status, consistent with previous studies carried out in sub-Saharan Africa [19, 26–28], and as documented in a multi-country analysis of DHS data [29].

This study however has some limitations. Foremost, the analysis draws on cross-sectional data; hence the causal nature of associations should be viewed with a certain caution. A second most important limitation of the study relates to the smaller sample size for individuals over 5 years compared to that of children 6–59 months, limiting the authors’ ability to potentially detect important differences and interactions between risk factors and transmission. Furthermore, the low proportion of males for this survey (34 %) may have triggered a gender-response bias, with consequences on the prevalence and associations found. The direction and magnitude of a possible bias remain unknown. Lastly, RDTs are limited in sensitivity to detect low density parasitaemia and their use may have led to an underestimation of the true proportion of people infected with *P. falciparum*. The underestimation may have differed with acquired immunity affecting the age pattern [30–33].

## Conclusions

For the most part, there was no evidence of an interaction between malaria infections and the risk factors with transmission intensity; however the associations with age and with fever in the last 2 weeks did vary significantly. The results also show that school-aged children are the least protected with ITN, across the different transmission settings, hence representing an important reservoir for infection. The observation of a shift in the peak age of risk for malaria to older groups is consistent with areas of low transmission and highlights the need for a more equal distribution of ITN in Kinshasa to target all age groups and not only the traditional high-risk group of young children.

## Abbreviations

HA: health area
HZ: health zone
ITN: insecticide-treated net
RDT: rapid diagnostic test
